# Associations Between Online Casual Sexual Behavior and HIV-Related Risk Behaviors Among Men Who Have Sex With Men in Southeast China: Cross-Sectional Study

**DOI:** 10.2196/71690

**Published:** 2025-10-17

**Authors:** Lin Chen, Zhongrong Yang, Wanjun Chen, Tingting Jiang, Xiaohong Pan

**Affiliations:** 1 Department of HIV/AIDS and STDs control and prevention, Zhejiang Provincial Center for Disease Control and Prevention, 3399 Binsheng Road, Binjiang District, Hangzhou, Zhejiang Province, 310052, China, 86 057187115190; 2 Department of HIV/TB control and prevention, Huzhou Center for Disease Control and Prevention, Huzhou, Zhejiang Province, China

**Keywords:** human immunodeficiency virus, men who have sex with men, sexual behavior on the internet, cross-sectional study, stimulants use

## Abstract

**Background:**

With the growing popularity and convenience of the internet, an increasing number of men who have sex with men (MSM) are seeking casual sexual partners online. However, the effect of online casual sexual behavior on other HIV-related risk behaviors remains unclear.

**Objective:**

This study aims to explore the characteristics of internet-based casual sexual behavior and its relationship with HIV-related risk behaviors among MSM.

**Methods:**

This cross-sectional study was conducted between June and December 2018 in 4 cities in Zhejiang Province, China. Peer-driven sampling was used for recruitment. Announcements were disseminated by 4 community-based organizations and 10 voluntary counseling and testing clinics online and offline. After informed consent, participants completed an electronic questionnaire covering demographic characteristics, casual sexual behaviors, HIV-related risk behaviors, and HIV prevention. SPSS (version 19.0; IBM Corp) was used to conduct chi-square tests, univariate and multivariate logistic regression analyses using a backward stepwise method based on the likelihood ratio test, and Poisson regression with robust variance to identify associations between finding casual sexual partners online and other risk behaviors. *P* values of <.05 were considered statistically significant.

**Results:**

In the past 6 months, 40.2% (302/751) of participants reported finding casual sexual partners online; 18.9% (142/751) reported finding casual sexual partners offline; 7.6% (57/751) reported having sexual intercourse with MSM without condoms after drinking alcohol; and 6.9% (52/751) reported condomless sex after using stimulants. Among those who found partners online, 62.5% (188/301) did so more than once per month and 39.5% (113/286) had more than one online sexual partner. In total, 39.3% (114/290) had sex with online partners at home and 10.1% (30/297) sought partners in other cities. Compared with participants who engaged in receptive anal intercourse (or both roles), those who engaged only in insertive intercourse reported a higher proportion of finding partners online more than once per month (72.7% vs 57.4%, *P*=.01), having more than 2 online sexual partners (52.1% vs 33.3%, *P*=.002), and conducting inconsistent condom use with online sexual partners (40.0% vs 25.8%, *P*=.01). Regression analysis showed that, compared with MSM who did not find partners online, those who did were more likely to report finding casual sexual partners offline (adjusted odds ratio [aOR] 9.398; 95% CI 5.956‐14.829), having sex without condoms after drinking alcohol (adjusted prevalence ratio [aPR] 1.788; 95% CI 1.062‐3.011), and having sexual intercourse without condoms after using stimulants (aPR 2.064; 95% CI 1.178‐3.617).

**Conclusions:**

Internet-based casual sexual behavior is increasingly common among MSM. Finding partners online was associated with offline partner-seeking and condomless use after alcohol or stimulant use. Future HIV prevention efforts should emphasize behavioral interventions tailored to MSM who use dating apps.

## Introduction

In recent years, men who have sex with men (MSM) have been one of the populations most seriously affected by HIV globally [1-3]. In China, the HIV-positive rate was the highest among MSM, compared with other high-risk populations (injecting drug users and commercial sex workers) in China [4,5]. In Zhejiang Province, nearly 40% of HIV-positive individuals acquired HIV through male-to-male sexual behavior. MSM have become a high-risk group for HIV infection, largely due to factors such as unprotected sex, multiple sexual partners, casual sex, and substance abuse [6,7]. These behaviors are closely tied to broader developments in social science and technology—particularly the rise of the internet, which has facilitated widespread online sexual behavior.

In recent years, smartphone apps such as Grindr (Grindr LLC), Jack’d (Lucid Dreams LLC), Blued (BlueCity Holdings Ltd), Manhunt (Online Buddies, Inc), Scruff (Perry Street Software, Inc), and Black Gay Chat (BGCLive, LLC) have become increasingly popular among MSM [8]. In China, Blued is the most widely used social app among MSM, with more than 30 million registered users nationwide. It is estimated that there are 409,000 MSM in Zhejiang Province and 8.28 million MSM in mainland China, accounting for roughly 1.732% (8,288,536/478,552,887) of all adult men aged 18-64 years in China [9]. Data show that 71.8% (18/252) of MSM report engaging in online sexual behavior, with this figure reaching 60% in China [10,11].

The internet has brought convenience and greater privacy to sexual activity among MSM, particularly for individuals with higher levels of education. However, the increased frequency of sexual encounters and the unknown HIV status of online partners have contributed to the ongoing spread of HIV and other sexually transmitted infections (STIs) within the MSM community [12]. A cohort study found that seeking sexual partners online was an independent risk factor for HIV infection [9]. This trend calls for urgent public health attention.

Regarding online casual sex, recent research has focused on the prevalence, characteristics, risk factors, and potential interventions related to internet-based sexual behaviors [13-15]. One study in India, for instance, compared current internet-related sexual behaviors between MSM and male-to-female transgender individuals [13]. However, the broader impact of online sex behavior on other HIV-related risk behaviors has often been overlooked. A systematic narrative review indicated that the existing literature does not clearly establish a link between online partner-seeking and condom use or STI status [16]. This gap highlights the need for more in-depth research in the field.

This study hypothesized that casual sexual behavior online influences HIV-related risk behaviors. Based on the literature, the following behaviors were selected as key outcomes potentially linked to HIV infection: offline casual sex, inconsistent condom use with MSM after alcohol consumption, and inconsistent condom use with MSM after stimulant use [17-19]. Compared with those who find partners online, individuals seeking partners offline report more sexual partners, a higher frequency of sexual activity, and lower rates of condom use [17]. In addition, alcohol and stimulant use may further reduce condom use and increase the likelihood of HIV infection [18,19]. One study found that 39.5% (230/582) of men reported using alcohol before sex in the past 3 months [18], and 40.7% (483/1186) reported stimulant use among MSM [20]. These 3 behaviors are closely associated with HIV transmission among MSM.

While a few studies have explored the relationship between online sex behavior and condom use, there remains a lack of research on condom use in the context of alcohol and stimulant use [21]. A cross-sectional study was conducted in 2018 in Zhejiang Province to gain a deeper understanding of the characteristics of online casual sex and its impact on other high-risk sexual behaviors. The findings aim to provide empirical evidence for future policy and strategy development.

## Methods

### Study Participants

This cross-sectional study was conducted between June and December 2018 among MSM in 4 cities—Hangzhou, Ningbo, Wenzhou, and Shaoxing in Zhejiang Province, China. The inclusion criteria were as follows: individuals who (1) had anal or oral intercourse with a male within the past 6 months, (2) were aged 18 years or older, (3) resided locally for more than 6 months, and (4) consented to participate in the study.

### Study Design and Data Collection

Participants were recruited through 4 local community-based organizations (CBOs) and 10 voluntary counseling and testing (VCT) clinics in Zhejiang Province, via venues for gay men and networks frequented by gay men. More than half of all MSM in the province resided in the cities of Hangzhou, Ningbo, Wenzhou, and Shaoxing. A peer-driven sampling method was used. First, CBOs posted recruitment advertisements in locations such as bathrooms, bars, and parks, as well as on online platforms, including social apps (Blued, WeChat [Tencent], and QQ [Tencent]) and social media. In addition, VCT clinics promoted the study in their physical offices and on their WeChat official accounts. Second, MSM already enrolled through CBOs or VCT clinics were encouraged to refer their peers.

To screen for eligibility, the participants answered 4 preliminary questions. After receiving training from either a CBO member or a physician, the participants scanned a QR code to access an electronic informed consent form. Those who selected “Agree” proceeded to complete the questionnaire.

The electronic questionnaire was developed using Wenjuanxing (Changsha Ranxing Information Technology Co, Ltd), a secure online survey platform that requires a username and password for access and data management. After its initial design, the questionnaire was pilot-tested with 5 respondents to refine its structure. Skip logic and mandatory responses were implemented to improve data quality. The final questionnaire included 39 items.

Participants received a gift valued at 30 RMB (approximately US $5) upon completion. To verify against duplicate entries, participants were asked to authorize their mobile phone numbers—no duplicates were identified. Five MSM were involved in designing the survey protocol and reviewing the questionnaire. Respondents were also able to review and revise their answers using a “Back” button.

The required sample size was calculated based on an estimated 40%‐60% rate of online sexual partners seeking online among MSM. Using PASS software version 11.0 (NCSS, LLC), the minimum sample size was determined to be 266 participants, assuming *α*=.1 and *β*=.1.

### Definition of Key Variables

Regarding demographic characteristics, participants reported their age, educational background, registered permanent residence, income, and sexual role. Age was calculated from birth dates provided in the questionnaire. Background was categorized into illiterate, primary school, junior middle school, high school, college, and postgraduate education. For logistic regression analysis, educational background was dichotomized as “high school or below” and “college or above.” Participants who reported having their registered in the local county or district were classified as “native.” Previous research has shown that individuals with a receptive sexual role are at higher risk of HIV infection than are individuals with an insertive role. In this study, sexual role was coded as “receptive or both” or “insertive only” for use in regression analyses and in characterizing individuals who seek partners online.

### Characteristics of HIV-Related Risky Behavior

Participants were asked, “Have you ever dated and had casual sexual intercourse with men you met on the internet, such as via Blued, WeChat, a chat room, or other platforms?” Those who answered “Yes” were defined as having “found casual sexual partners online.” Participants were also asked, “Have you ever had casual sex intercourse with men you met in a bar, park, bathing pool, or other place in the past 6 months?” Those who answered “Yes” were categorized as having “found sexual partners offline.”

To assess condom use after alcohol consumption, participants were asked: “Have you ever engaged in sexual behavior without a condom after drinking in the last 6 months?” Those who answered “Yes” were defined as having engaged in the relevant risk behavior.

For stimulant use, participants were asked: “Have you ever engaged in sexual behavior without a condom after using stimulants, such as ‘rush poppers’ or ‘zero capsules,’ in the last 6 months?” Again, participants answering “Yes” were categorized as having engaged in this risk behavior. The main components of “rush poppers” and “zero capsules” are various nitrite esters and dimethyltryptamine (5-MeO-DiPT), respectively. Both stimulants are popular among MSM and enhance sexual stimulation.

### Characteristics of Finding Sexual Partners Online

The variables used to describe the characteristics of finding casual partners online were the frequency of finding partners online (once a month or more often), number of online sexual partners (one or more), place where casual sex occurred (home, karaoke lounge, club, or hotel), city where the encounter took place (local city or other city), age of the online sexual partner (0‐30 or >30 years), and condom use with online sexual partners (every time, sometimes, or never).

In the chi-square test, the place of sexual intercourse was recorded as “hotel or karaoke lounge or club” or “home.” Condom use was recorded into “consistent condom use” (reported use every time) and “inconsistent condom use” (reported use sometimes or never). All variables refer to behaviors within the previous 6 months.

The selection of variables related to finding sexual partners online was based on their importance in the literature and frequency of appearance in previous research reports.

### Other Confounders

Participants were asked the following question regarding HIV intervention: “Have you ever seen any HIV–related information or propaganda on a dating app, such as Blued, WeChat, Aloha, etc.?” Responding “Yes” was defined as “acquired HIV information from a dating app.” For logistic regression, responses were dichotomized as “No” and “Yes.”

Another variable was assessed through the question: “Have you seen a sexually explicit video on the internet in the last 6 months?” Response options included: “3‐7 times per week,” “1‐2 times per week,” “2 times per month,” “1 time per month,” “less than 1 time per month,” and “Never.” For logistic regression analysis, this variable was also dichotomized as “No” and “Yes.”

### Data Analysis

Data were analyzed using SPSS software (version 19.0; IBM Corp). Descriptive analyses were conducted to summarize demographic characteristics and risky sexual behaviors among all MSM participants. The Pearson chi-square tests were used to examine differences in characteristics of web-based casual sexual behavior between groups with different educational backgrounds and sexual roles. Univariate and multivariate logistic regression analyses using a backward stepwise method based on the likelihood ratio test were performed to assess the relationship between finding casual sexual partners online and finding casual sexual partners offline. Poisson models with robust variance were performed to evaluate the relationship between finding casual sexual partners online and sexual intercourse with MSM without condoms after drinking alcohol, as well as sexual intercourse with MSM without condoms after using stimulants. All multivariate models were adjusted for 4 confounders: education level (0=high school or below, 1=college or above), sexual role (0=receptive or both, 1=insertive only), acquisition of HIV information from a dating app (0=no, 1=yes), and viewing sexually explicit videos on the internet (0=no, 1=yes). Missing data were excluded from the analysis but are reported in the tables. A *P* value of <.05 was considered statistically significant, and all hypothesis tests were 2-sided.

Ethical Considerations

All procedures in this study were approved by the Ethics Committee of the Zhejiang Provincial Center for Disease Control and Prevention (approval number: 2018‐033). Electronic informed consent was obtained from all participants. Before completing the questionnaire, respondents were informed—either in person by CBO staff or by phone—about the purpose, estimated duration, survey content, and data confidentiality. The informed consent form specified that deidentified data could be used for secondary analysis without the need for reconsent. To ensure confidentiality, phone numbers were anonymized and permanently deleted from the dataset after verification. Each respondent will receive a gift worth US $4, and CBOs will get a reward of US $7 for completing the questionnaire survey of each respondent.

## Results

**Figure 1.**
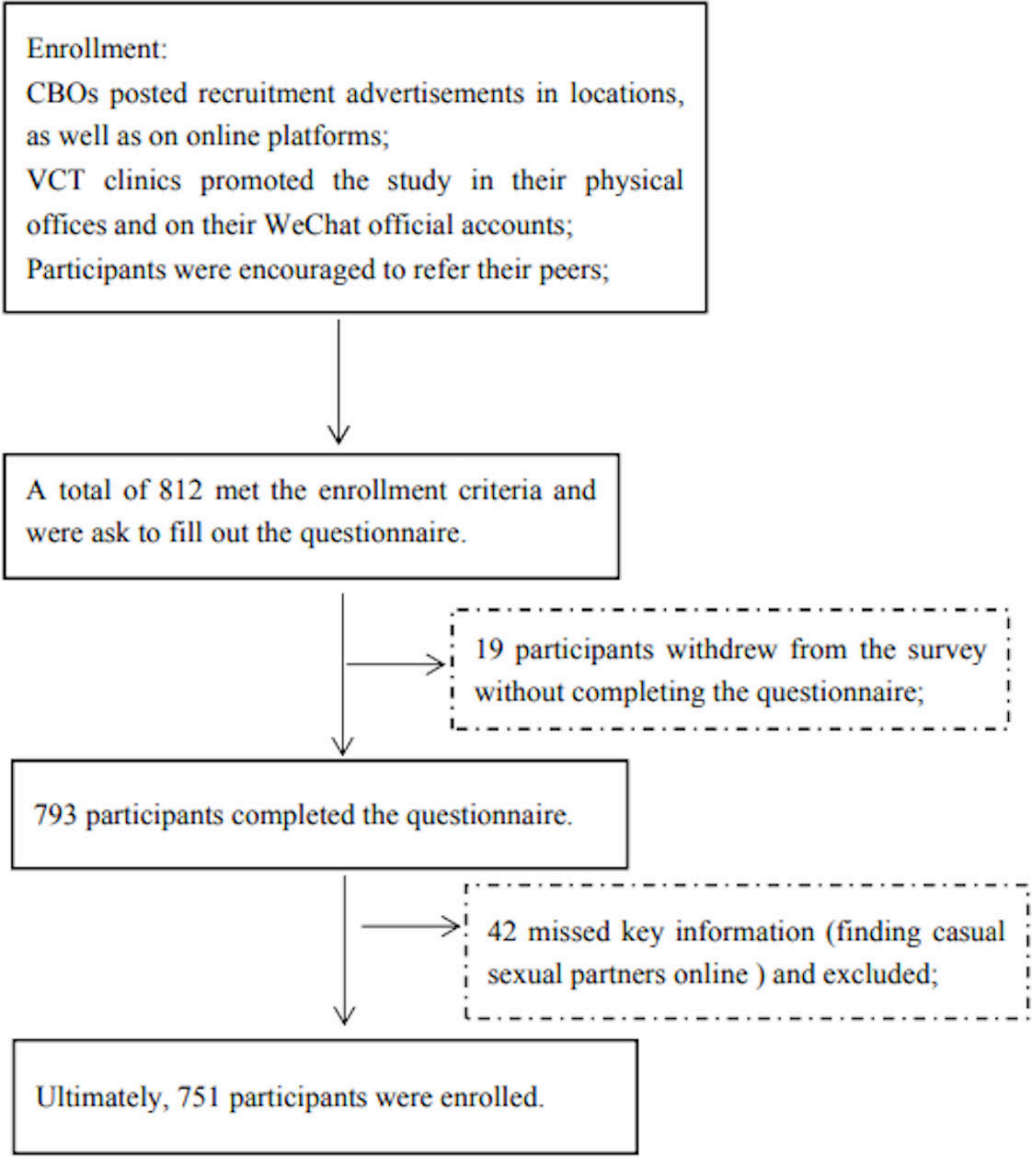
Recruitment process flow chart for participants. CBO: community-based organization; VCT: voluntary counseling and testing.

In total, 812 individuals met the inclusion criteria. Of these, 19 did not complete the questionnaire, resulting in 793 (97.7%) valid initial responses being included. After excluding 42 participants with missing key information, the final sample included 751 participants. The recruitment procedure is shown in Figure 1.

Among the 751 participants, 24.1% (181/751) were aged 35 years or older, 61.9% (465/751) had a college education or higher, and 63.9% (480/751) had ever engaged in a receptive sexual role. In the past 6 months, 67.0% (503/751) reported having acquired HIV information from dating apps and 78.7% (591/751) had viewed sexually explicit videos on the internet (Table 1).

**Table 1.** Sociodemographic characteristics, HIV-related risk behaviors, intervention exposure, and associations with other HIV-related risk behaviors. Data are presented as n (%).

Variables	All MSM^a^ (N=751)	Found casual sexual partners offline (n=142)	Chi-square (*df*)	*P* value	Sexual intercourse with MSM without condoms after drinking alcohol (n=57)	Chi-square (*df*)	*P* value	Sexual intercourse with MSM without condoms after using stimulants (n=52)	Chi-square (*df*)	*P* value
Age (years)			5.133 (2)	.08		0.094 (2)	.95		0.159 (2)	.92
18‐24	241 (32.1)	45 (18.7)			18 (7.5)			18 (7.5)		
25‐34	329 (43.8)	53 (16.1)			26 (7.9)			22 (6.7)		
≥35	181 (24.1)	44 (24.3)			13 (7.2)			12 (6.6)		
Education level			11.831 (1)	.001		8.530 (1)	.003		0.003 (1)	.09
High school or below	286 (38.1)	72 (25.2)			32 (11.2)			20 (7.0)		
College or above	465 (61.9)	70 (15.1)			25 (5.4)			32 (6.9)		
Sexual role			4.359 (1)	.03		0.169 (1)	.68		0.004 (1)	.95
Receptive or both	480(63.9)	80(16.7)			35(7.3)			33(6.9)		
Insertive only	271(36.1)	62(22.9)			22(8.1)			19(7.0)		
Acquired HIV information from a dating app			8.708 (1)	.003		0.407 (1)	.52		0.133 (1)	.72
No	248 (33.0)	32 (12.9)			21 (8.5)			16 (6.5)		
Yes	503 (67.0)	110 (21.9)			36 (7.2)			36 (7.2)		
Watched sexually explicit videos on the internet			0.003 (1)	.95		1.119 (1)	.29		3.194 (1)	.07
No	160 (21.3)	30 (18.8)			9 (5.6)			6 (3.8)		
Yes	591 (78.7)	112 (19.0)			48 (8.1)			46 (7.8)		
Found casual sexual partners online			112.861 (1)	<.001		5.1531 (1)	.02		8.826 (1)	.003
No	449 (59.8)	29 (6.5)			26 (5.8)			21 (4.7)		
Yes	302 (40.2)	113 (37.4)			31 (10.3)			31 (10.3)		

a MSM: men who have sex with men.

Within the past 6 months, 40.2% (302/751) of the participants reported having found casual sexual partners online; 18.9% (142/751) had found casual partners at bars, bathrooms, or parks; 7.6% (57/751) reported having sexual intercourse with MSM without condoms after drinking alcohol; and 6.9% (52/751) reported having sex without condoms after using stimulants (Table 1).

The chi-square test results showed statistically significant differences in the prevalence of 3 HIV-related risk behaviors between those who found partners online and those who did not (*P*<.05): finding casual sexual partners offline (37.4%, 113/302 vs 6.5%, 29/449; *P*<.001), sexual intercourse with MSM without a condom after drinking alcohol (10.3%, 31/302, vs 5.8%, 26/449; *P*=.02), and sexual intercourse without a condom after using stimulants (10.3%, 31/302 vs 4.7%, 21/449; *P*=.003; Table 1). Among MSM who reported finding casual sexual partners online, 62.5% (188/301) met partners more than once per month; 39.5% (113/286) had more than one online sexual partner; 60.7% (114/290) had sex with partners at a hotel, karaoke lounge, or club; 89.9% (267/297) found casual partners in the local city; 72.2% (213/298) preferred partners younger than 30 years; and 69.4% (206/297) reported using condoms every time with online sexual partners. The most common partner selection preferences were body shape and age (Table 2).

**Table 2.** Frequency, condom use, and locations of online sexual encounters among men who have sex with men who sought sexual partners online in the past 6 months.

Variables	N (%)	Sexual role		Chi-square (*df*)	*P* value
		Receptive anal or both, n (%)	Inserted anal only, n (%)		
Frequency of finding partners online (time per month)				6.634 (1)	.01
1	113 (37.5)	86 (42.6)	27 (27.3)		
>1	188 (62.5)	116 (57.4)	72 (72.7)		
Missing	1				
Number of online sexual partners				9.327 (1)	.002
2	173 (60.5)	128 (66.7)	45 (47.9)		
>2	113 (39.5)	64 (33.3)	49 (52.1)		
Missing	16				
Place where casual sexual intercourse occurred				0.361 (1)	.55
Hotel or karaoke lounge or club	176 (60.7)	116 (59.5)	60 (63.2)		
Home	114 (39.3)	79 (40.5)	35 (36.8)		
Missing	12				
City in which casual sexual intercourse occurred				0.167 (1)	.68
Local city	267 (89.9)	177 (89.4)	90 (90.9)		
Other city	30 (10.1)	21 (10.6)	9 (9.1)		
Missing	5				
Age of online sexual partners				0.230 (1)	.63
0‐30 years	213 (71.5)	144 (72.4)	69 (69.7)		
>30 years	85 (28.5)	55 (27.6)	30 (30.3)		
Missing	4				
Condom use with online sexual partners				6.662 (1)	.01
Consistent	206 (69.4)	147 (74.2)	59 (59.6)		
Inconsistent	91 (30.6)	51 (25.8)	40 (40.0)		
Missing	5				

Compared with participants who engaged in receptive anal intercourse (or both roles), those who engaged only in insertive intercourse reported a higher proportion of finding partners online more than once per month (72.7%, 72/99 vs 57.4%, 116/202; *P*=.01), having more than 2 online sexual partners (52.1%, 49/94 vs 33.3%, 64/192; *P*=.002), and inconsistent condom use with online sexual partners (40.0%, 40/99 vs 25.8%, 51/198; *P*=.01; Table 2).

In the univariate and multivariate logistic regression analyses, the relationships between finding casual sexual partners online and other risky sexual behaviors were evaluated, after controlling for education (0=High School and under, 1=College and above), sex role (0=Receptive or both, 1= Insertive only), acquisition of HIV information from a dating app (0=No, 1=Yes), and watching obscene videos on the internet (0=No, 1=Yes). Compared to MSM who did not find partners online, those who did reported a higher likelihood of finding casual sexual partners offline (adjusted odds ratio [aOR] 9.398; 95% CI 5.956‐14.829), having sexual intercourse without condoms after drinking alcohol (adjusted prevalence ratios [aPR] 1.788; 95% CI 1.062‐3.011), and having sexual intercourse without condoms after using stimulants (aPR 2.064; 95% CI 1.178‐3.617; Table 3).

**Table 3.** Univariate and multivariate logistic regression analyses of associations between finding casual sexual partners online and 3 HIV-related risk behaviors in the past 6 months among men who have sex with men.

Variables	Found casual sexual partners offline		Sexual intercourse with MSM^a^ without condoms after drinking alcohol		Sexual intercourse with MSM without condoms after using stimulants	
Crude OR^b^ (95% CI)	Adjusted OR (95% CI)	Crude PR^c^ (95% CI)	Adjusted PR (95% CI)	Crude PR (95% CI)	Adjusted PR (95% CI)
Education	0.527 (0.364‐0.762)	0.460 (0.304‐0.697)	0.481 (0.285‐0.811)	0.477 (0.283‐0.805)	0.986 (0.564‐1.724)	—^d^
Sex role	1.483 (1.023‐2.150)	1.723 (1.132‐2.623)	1.113 (0.653‐1.898)	—	1.018 (0.579‐1.790)	—
Acquired HIV information from dating app	1.889 (1.232‐2.896)	1.829 (1.148‐2.918)	0.845 (0.493‐1.448)	—	1.112 (0.617‐2.003)	—
Watched sexually explicit videos on the internet	1.013 (0.648‐1.584)	—	1.444 (0.708‐2.943)	—	2.079 (0.888‐4.868)	—
Found casual sexual partners online	8.659 (5.562‐13.480)	9.398 (5.956‐14.829)	1.773 (1.053‐2.985)	1.788 (1.062‐3.011)	2.202 (1.265‐3.832)	2.064 (1.178‐3.617)

a MSM: men who have sex with men.

b OR: odds ratio.

c PR: prevalence ratio.

d Not available.

## Discussion

### Principal Findings

With the growing popularity of the internet, online casual sexual behaviors have become increasingly common. In this study, the rate of finding partners online for casual sexual behavior was nearly 40%, more than twice the rate of offline sex encounters. This may be attributed to the convenience and anonymity of the internet, which can facilitate the occurrence of casual sexual activity. Notably, the rate of online casual sex in this study was higher than that in Mexico but lower than that in the United States and India [13,21,22]. These differences may be linked to the earlier and broader adoption of internet technologies, as well as social culture.

When examining behaviors associated with increased risk of STI or HIV transmission, MSM with online sexual partners were found in other studies to report a greater number of partners, and higher proportions reported exchanging sex for money or drugs and having sex with partners who themselves had multiple partners [21]. This may represent one of the biggest challenges to HIV prevention in China over the next decade and deserves heightened attention.

Existing research exploring the complex dynamics of online casual sex among MSM remains limited, with only a few studies available [23]. This study contributes by outlining the demographic and behavioral characteristics of MSM and their online sexual partners. Notably, not all encounters took place in hotels; approximately half occurred at home, possibly because of cost-saving considerations. This shift complicates HIV prevention efforts because traditional strategies—such as distributing condoms and educational materials in hotels—may not be as effective in private domestic settings [24]. Data also indicate that a small proportion of MSM engage in online sexual activity while traveling to other regions. This includes individuals with stable partners who travel for work, as well as single individuals traveling for leisure. The increasing availability of mobile technology has linked mobility with heightened sexual risk behavior [25]. These findings raise questions about the accessibility and timeliness of intervention services, such as postexposure prophylaxis and HIV testing, for individuals in transit or away from home.

We also found that approximately one-third of the participants reported having online sexual partners aged 30 years or older. Compared with other populations, MSM are younger when they become infected with HIV [26]. The older a sexual partner is, the higher the probability that they are living with HIV. Seeking older sexual partners may increase HIV risk, particularly among those new to MSM social networks or those engaging in receptive anal intercourse—groups that warrant focused attention in HIV prevention strategies.

Regarding condom use, nearly one-third of participants reported inconsistent condom use with online sexual partners. This rate was especially high among those who reported only insertive anal intercourse and those with a high school education or below. Though the rate of inconsistent condom use in this study was lower than that reported in some other countries [27], it remains a concern. One study found that MSM were less likely to engage in condomless anal sex with partners met online compared with those met offline, possibly because they were more likely to ask about HIV status and testing history beforehand [28]. Nonetheless, the rate of condomless sex continues to represent a key driver of HIV transmission in this population and should not be overlooked.

Many researchers have found that MSM who meet their partners online are more likely to engage in risky sexual behaviors, such as receptive anal sexual partners and reporting condomless receptive anal sex, past 6 months of illicit drug use in the previous 6 months, and having sex with anonymous partners whose HIV status is unknown [17,29]. One study found that HIV-positive MSM had significantly more partners whom they had met online than did HIV-negative MSM [13]. In this study, after adjusting for confounders, we found persistent associations between finding casual sexual partners online and 3 HIV-related risk behaviors: finding casual sexual partners offline, engaging in sexual activity without condoms after drinking alcohol, and engaging in sexual activity without condoms after using stimulants. These findings support existing evidence that online sexual behavior is linked to increased HIV risk among MSM.

The effects of drinking alcohol and stimulant use on HIV infection risk have been well-documented [18,19]. Rush poppers have become one of the most popular stimulants among MSM in China, used for both pain relief and sexual pleasure. Reports suggest that 20%‐30% of MSM in China use rush poppers [11,30]. Users were more likely to have multiple sexual partners, casual partners, or STIs [31]. A 2018 US study found that 59.9% of MSM reported alcohol or illicit drug use before or during sex [10]. Both an increased number of insertive and receptive partners and substance use during sex were associated with higher odds of seeking partners online [10].

This study implies that online sexual behavior may play a driving role in the occurrence of other risk behaviors. For example, individuals who engage in sex with unfamiliar online partners—particularly receptive partners—may be more likely to use sexual stimulants, leading to impaired judgment or reduced control over condom use. In the case of alcohol, some MSM may intentionally meet for drinks or meals before sex to reduce awkwardness or build comfort, which may contribute to condomless sex. These patterns suggest that HIV-related risk behaviors tend to cluster among MSM—especially those at highest risk of infection—who should be identified as a priority population for receiving high-quality, targeted interventions [32]. Cohort studies are needed to further clarify these relationships. Although it is not feasible to prevent online partner-seeking behavior, risk-reduction strategies and services can be strengthened and tailored to reduce harms when such behaviors occur.

This study has some limitations. First, the cross-sectional design limits our ability to determine causal relationships between online partners seeking and other risk behaviors. Second, the peer-driven sampling method may lead to sampling bias and thus fail to represent the real MSM population. To reduce such bias, we made efforts to disseminate recruitment information through various channels to decrease the homogeneity of the respondents. In addition, we identified repeated participants using their mobile phone numbers. Third, data were collected via self-administered online questionnaires, which may have introduced information bias. However, we implemented strict quality control procedures, such as phone follow-ups and questionnaire checks, to help minimize this bias. Future multicenter cohort studies with larger sample sizes are needed to confirm and expand on these findings.

### Conclusion

Finding sexual partners online is common among MSM in Zhejiang Province. Most MSM seek partners online more than once per month, and the majority of their partners are younger than 30 years of age. Hotels, lounges, and clubs are the main locations for sexual encounters. MSM with a high school education or below were more likely to report condomless sexual behavior. Those who engaged in insertive anal intercourse only reported higher frequencies of online partner-seeking and a greater number of online partners. Among MSM, there were clear associations between finding sexual partners online and other HIV-related risk behaviors, including finding partners offline, engaging in condomless sex after drinking alcohol, and engaging in condomless sex after using stimulants. These findings underscore the need for targeted, integrated prevention strategies focused on MSM populations who use online platforms to seek sexual partners.

## Abbreviations

aOR: adjusted odds ratio
aPR: adjusted prevalence ratio
CBO: community-based organization
MSM: men who have sex with men
STI: sexually transmitted infection
VCT: voluntary counseling and testing
